# Disabling musculoskeletal pain in working populations: Is it the job, the person, or the culture?

**DOI:** 10.1016/j.pain.2013.02.008

**Published:** 2013-06

**Authors:** David Coggon, Georgia Ntani, Keith T. Palmer, Vanda E. Felli, Raul Harari, Lope H. Barrero, Sarah A. Felknor, David Gimeno, Anna Cattrell, Consol Serra, Matteo Bonzini, Eleni Solidaki, Eda Merisalu, Rima R. Habib, Farideh Sadeghian, M. Masood Kadir, Sudath S.P. Warnakulasuriya, Ko Matsudaira, Busisiwe Nyantumbu, Malcolm R. Sim, Helen Harcombe, Ken Cox, Maria H. Marziale, Leila M. Sarquis, Florencia Harari, Rocio Freire, Natalia Harari, Magda V. Monroy, Leonardo A. Quintana, Marianela Rojas, Eduardo J. Salazar Vega, E. Clare Harris, Sergio Vargas-Prada, J. Miguel Martinez, George Delclos, Fernando G. Benavides, Michele Carugno, Marco M. Ferrario, Angela C. Pesatori, Leda Chatzi, Panos Bitsios, Manolis Kogevinas, Kristel Oha, Tuuli Sirk, Ali Sadeghian, Roshini J. Peiris-John, Nalini Sathiakumar, A. Rajitha Wickremasinghe, Noriko Yoshimura, Helen L. Kelsall, Victor C.W. Hoe, Donna M. Urquhart, Sarah Derrett, David McBride, Peter Herbison, Andrew Gray

**Affiliations:** a Medical Research Council Lifecourse Epidemiology Unit, University of Southampton, UK; b School of Nursing, University of São Paulo, São Paulo, Brazil; c Corporación para el Desarrollo de la Producción y el Medio Ambiente Laboral – IFA (Institute for the Development of Production and the Work Environment), Quito, Ecuador; d Department of Industrial Engineering, School of Engineering, Pontificia Universidad Javeriana, Bogotá, Colombia; e Southwest Center for Occupational and Environmental Health, The University of Texas Health Science Center at Houston School of Public Health, Houston, Texas, USA; f Center for Disease Control and Prevention/National Institute for Occupational Safety and Health, Atlanta, GA, USA; g Medical Research Council Social, Genetic and Developmental Psychiatry Centre, Institute of Psychiatry, Kings College, London, UK; h Center for Research in Occupational Health (CiSAL), Universitat Pompeu Fabra, Barcelona, Spain; i CIBER of Epidemiology and Public Health, Barcelona, Spain; j Occupational Health Service, Parc de Salut MAR, Barcelona, Spain; k Epidemiology and Preventive Medicine Research Center, University of Insubria, Varese, Italy; l Department of Social Medicine, Medical School, University of Crete, Heraklion, Greece; m Department of Public Health, University of Tartu, Estonia; n Department of Environmental Health, Faculty of Health Sciences, American University of Beirut, Beirut, Lebanon; o Department of Occupational Health, Faculty of Health, Shahroud University of Medical Sciences, Shahroud, Iran; p Department of Community Health Sciences, Aga Khan University, Karachi, Pakistan; q Department of Medical Education and Health Sciences, Faculty of Medical Sciences, University of Sri Jayewardenepura, Gangodawila, Nugegoda, Sri Lanka; r Clinical Research Centre for Occupational Musculoskeletal Disorders, Kanto Rosai Hospital, Kawasaki, Japan; s National Institute for Occupational Health, National Health Laboratory Service, Johannesburg, South Africa; t Faculty of Health Sciences, University of Witwatersrand, Johannesburg, South Africa; u Department of Epidemiology and Preventive Medicine, School of Public Health and Preventive Medicine, Monash University, Melbourne, Victoria, Australia; v Department of Preventive and Social Medicine, University of Otago, Dunedin, New Zealand; w School of Nursing of Ribeirão Preto, University of São Paulo, São Paulo, Brazil; x Federal University of Paraná, Curitiba-PR, Brazil; y Institute for Studies on Toxic Substances (IRET), National University of Costa Rica, Heredia, Costa Rica; z Department of Clinical Sciences and Community Health, Università degli Studi di Milano, Milan, Italy; aa Fondazione Ca’ Granda Ospedale Maggiore Policlinico, Milan, Italy; ab Department of Psychiatry, Medical School, University of Crete, Heraklion, Greece; ac Centre for Research in Environmental Epidemiology (CREAL), Barcelona, Spain; ad IMIM (Hospital del Mar Research Institute), Barcelona, Spain; ae National School of Public Health, Athens, Greece; af North Estonia Medical Centre, Tallinn, Estonia; ag Põlva Hospital, Põlva, Estonia; ah Klinikum Leverkusen, Leverkusen, Germany; ai Department of Physiology, Faculty of Medical Sciences, University of Sri Jayewardenepura, Gangodawila, Nugegoda, Sri Lanka; aj Section of Epidemiology and Biostatistics, School of Population Health, Faculty of Medical and Health Sciences, University of Auckland, New Zealand; ak Department of Epidemiology, School of Public Health, University of Alabama at Birmingham, USA; al Faculty of Medicine, University of Kelaniya, Kelaniya, Sri Lanka; am Department of Joint Disease Research, University of Tokyo, Tokyo, Japan; an Centre for Occupational and Environmental Health, Department of Social and Preventive Medicine, Faculty of Medicine, University of Malaya, Kuala Lumpur, Malaysia; ao Injury Prevention Research Unit, Department of Preventive and Social Medicine, University of Otago, Dunedin, New Zealand

**Keywords:** Low back, Forearm, Pain, International, Socioeconomic, Psychosocial

## Abstract

To compare the prevalence of disabling low back pain (DLBP) and disabling wrist/hand pain (DWHP) among groups of workers carrying out similar physical activities in different cultural environments, and to explore explanations for observed differences, we conducted a cross-sectional survey in 18 countries. Standardised questionnaires were used to ascertain pain that interfered with everyday activities and exposure to possible risk factors in 12,426 participants from 47 occupational groups (mostly nurses and office workers). Associations with risk factors were assessed by Poisson regression. The 1-month prevalence of DLBP in nurses varied from 9.6% to 42.6%, and that of DWHP in office workers from 2.2% to 31.6%. Rates of disabling pain at the 2 anatomical sites covaried (*r* = 0.76), but DLBP tended to be relatively more common in nurses and DWHP in office workers. Established risk factors such as occupational physical activities, psychosocial aspects of work, and tendency to somatise were confirmed, and associations were found also with adverse health beliefs and group awareness of people outside work with musculoskeletal pain. However, after allowance for these risk factors, an up-to 8-fold difference in prevalence remained. Systems of compensation for work-related illness and financial support for health-related incapacity for work appeared to have little influence on the occurrence of symptoms. Our findings indicate large international variation in the prevalence of disabling forearm and back pain among occupational groups carrying out similar tasks, which is only partially explained by the personal and socioeconomic risk factors that were analysed.

## Summary

Large international variation in the prevalence of disabling forearm and low back pain was only partially explained by established personal and socioeconomic risk factors.

## 1 Introduction

In Europe, musculoskeletal disorders, especially of the back and upper limb, are the biggest single cause of incapacity for work, with direct costs amounting to between 0.5% and 2% of gross domestic product [1]. In many cases they are attributed to mechanical stresses from occupational activities such as heavy lifting and repetitive movements of the wrist and hand, and this has prompted legislation requiring employers to ensure that methods of work are ergonomically sound [8,9].

Unlike many occupational hazards, however, back and arm pain are not a simple consequence of harmful physical exposures. There is good evidence from observational studies that they are also associated with, and predicted by, psychological risk factors such as low mood and somatising tendency (a general tendency to worry about common somatic symptoms) [11,12,18,19]. In addition, they have been linked, although less consistently, with various psychosocial aspects of work, such as low control, support, and job satisfaction [13]. Moreover, there are indications that their prevalence varies among countries, and within countries over time, in a way that cannot be explained by known causes [4,14]. This has led to the hypothesis that their occurrence, and especially their chronicity and resultant disability, are strongly influenced by adverse health beliefs and expectations, acting through a nocebo effect [4].

If correct, this would have important practical implications. Good ergonomic practice reduces physical stresses that can trigger symptoms, and makes tasks easier and more comfortable. However, if presented in the wrong way, it could also promote an exaggerated belief among workers that they are exposed to serious risk of injury, and thereby cause a paradoxical increase in symptoms and disability. An effect of this sort might explain why randomised controlled trials of ergonomic interventions to prevent low back pain have failed to show benefit [7].

Another reason for differences in prevalence among countries might be that rates of disabling musculoskeletal pain are influenced by systems of compensation for work-related illness and injuries, and of financial support for health-related incapacity for work. The possibility of financial benefits from a health problem could be a subconscious stimulus to illness that would not otherwise occur.

The Cultural and Psychosocial Influences on Disability (CUPID) study is an international, multi-centre epidemiological investigation that was established to explore the contribution of culturally determined health beliefs and other psychosocial and economic risk factors to the disability arising from common musculoskeletal complaints [5]. We here present findings on low back and wrist/hand pain, in which we compare the frequency of disabling symptoms among groups of workers carrying out similar physical activities in different cultural and socioeconomic environments, and assess the extent to which variations in prevalence can be explained by putative risk factors, including health beliefs and social security provisions.

## 2 Methods

The study was conducted by teams of investigators in each of 18 countries (Table 1), data then being forwarded for analysis by a coordinating group in Southampton, UK. Ethical approval was provided by the relevant research ethics committee in each country [5].

### 2.1 Study sample

Data collection was carried out during 2006–2011, using methods that have been reported in detail elsewhere [5]. The study sample comprised 47 occupational groups (1–4 per country; see Table 1), which fell into 3 categories: nurses (including nursing assistants), office workers, and “other workers” (mainly jobs entailing repetitive tasks with the hands or arms, postal workers being the most common). All participants were aged 20–59 years, and all had been in their current job for at least 12 months. The aim was to recruit at least 200 workers in each occupational group, which would be more than adequate to detect differences in the prevalence of symptoms and disability of the magnitude that was anticipated.

### 2.2 Ascertainment of exposures and outcomes

Information about musculoskeletal symptoms and personal risk factors was elicited through a standardised questionnaire, either at interview (25 occupational groups), by self-administration (18 groups), or a combination of the 2 (4 groups). The questionnaire was first drafted in English, and then translated into local languages where necessary. The accuracy of translation was checked by independent back-translation, with subsequent amendment if required.

Among other things, the questionnaire asked about: sex; age; smoking status; hours worked per week (classified as <50 or 50+); occupational lifting (whether an average working day entailed lifting weights of 25 kg or more by hand); whether an average working day entailed use of a keyboard for >4 h in total, and/or other tasks involving repeated movements of the wrists or fingers for >4 h in total; various psychosocial aspects of work (incentives from piecework or bonuses; time pressure; lack of choice in what work is done, how, and when; lack of support from colleagues or supervisor/manager; job dissatisfaction; and perceived job insecurity if off work for 3 months with illness); somatising tendency; mental health; adverse beliefs about musculoskeletal pain in the low back and arm (work-relatedness, prognosis, and effects of physical activity); whether the participant had heard of the term “repetitive strain injury” (RSI) or an equivalent; and about pain in the past month in the low back and wrist/hand (either or both sides).

Questions about somatising tendency were taken from the Brief Symptom Inventory [6], and subjects were classified according to the number of symptoms from a total of 5 (faintness or dizziness, pains in the heart or chest, nausea or upset stomach, trouble getting breath, hot or cold spells) that had been at least moderately distressing in the past week. Questions about mental health came from the relevant domain of the Short Form-36 questionnaire [20], and scores were classified to approximate thirds of the distribution in the full study sample (denoted good, intermediate, or poor).

In the ascertainment of musculoskeletal pain, the anatomical areas of interest were depicted in diagrams. For each site (low back and wrist/hand), subjects were asked whether they had experienced pain lasting for longer than a day at any time during the past month, and if so, whether during this time, the pain had made it difficult or impossible to carry out any of a specified list of everyday activities (for low back: getting dressed, doing normal jobs around the house, or cutting toe nails; for wrist/hand: getting dressed, doing normal jobs around the house, writing, locking/unlocking doors, or opening bottles/jars/taps). Pain was classed as disabling if it had made any of these activities difficult or impossible.

In addition to personal risk factors, we also ascertained a number of risk factors that operated at the level of the occupational group. Some of these were derived from the prevalence of personal characteristics within the relevant group as established from the questionnaire: the prevalence of knowing someone outside work with low back pain and with arm pain; the prevalence of adverse beliefs about musculoskeletal pain in the low back and arm; and the prevalence of having heard of the term “repetitive strain injury” (RSI) or an equivalent. Other group-level risk factors were provided by the local investigators in each participating country: the unemployment rate in the community from which the occupational group came; whether social security support was available for members of the community who were unemployed; the extent to which employees were eligible for pay during sickness absence; whether workers were entitled to compensation for work-related low back or wrist/hand disorders; whether special financial support was available in cases of ill-health retirement; whether a fee had to be paid to see a doctor in primary care; and whether workers had access to an occupational health service.

### 2.3 Statistical analysis

Statistical analysis was carried out with Stata version 12.1 software (StataCorp LP, College Station, TX, USA), and focused on 2 outcomes: disabling low back pain (DLBP) in the past month and disabling wrist/hand pain (DWHP) in the past month. We first compared the prevalence of these 2 outcomes across occupational groups. We then explored personal risk factors, using generalised linear latent and mixed models to fit random-effects Poisson regression models with robust standard errors. We employed 2-level models with individuals clustered by occupational group. For each outcome, the analysis incorporated all potentially relevant risk factors in a single model, and associations were summarised by prevalence rate ratios (PRRs) with associated 95% confidence intervals (95% CIs).

Next, we compared the observed counts of cases of disabling musculoskeletal pain in each occupational group with the numbers that would have been expected based on their distribution of individual-level risk factors and the overall prevalence of the 2 outcomes in the full study sample.

We then extended our Poisson regression models to include each group-level risk factor in turn, along with all of the personal risk factors. Those group-level risk factors, which showed statistically significant (*P* < 0.05) associations when examined separately, were re-analysed in a further model, together with all personal risk factors. Finally, we recalculated expected counts of cases by occupational group, taking into account significant group-level risk factors as well as personal risk factors, and again made comparison with the observed counts. To check what proportion of the residual variation was explained by systematic differences among countries, we derived estimates of between-country variance and additional variance among occupational groups from 3-level Poisson regression models that allowed for clustering by country as well as by occupational group.

## 3 Results

The response rate among those invited to take part in the study exceeded 80% in 33 of the 47 occupational groups, but was <50% in 5 groups. Usable questionnaires were obtained from a total of 12,426 participants, the number by occupational group ranging from 92 to 1018. The demographic characteristics of the study sample and the distributions of exposure to risk factors by occupational group have been described in detail elsewhere [5]. As expected, a high proportion of office workers (>80% in all but one group) reported using a keyboard for longer than 4 h per day, whereas manual lifting of weights ⩾25 kg was most common in nurses.

**Fig. 1:**
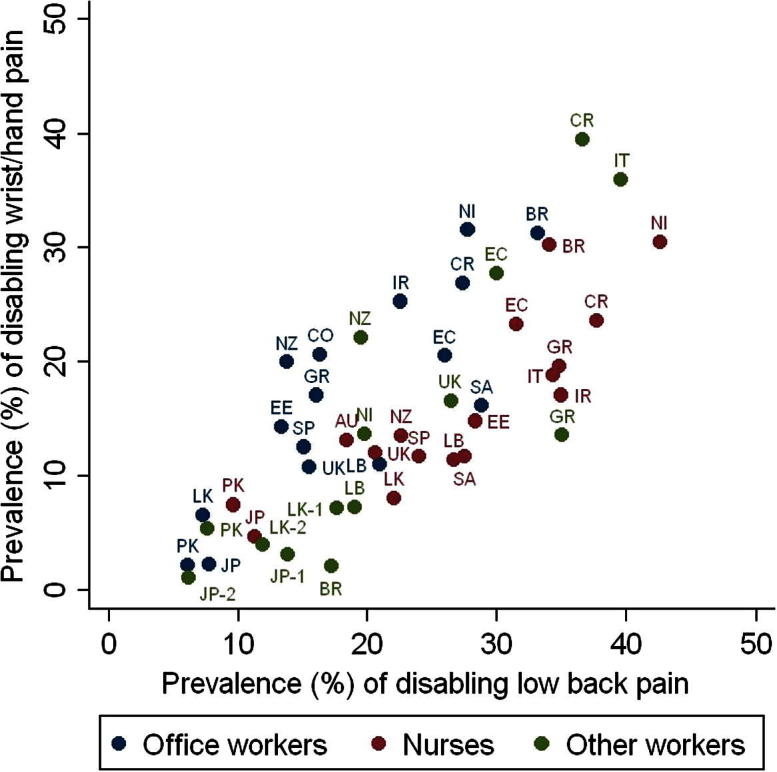
One-month prevalence of disabling low back and wrist/hand pain by occupational group.

Fig. 1 shows the 1-month prevalence of disabling low back and wrist/hand pain by occupational group. Overall, DLBP was reported by 22.0% of participants, and DWHP by 14.4%. However, there was marked variation among occupational groups (geometric SDs 1.68 for DLBP and 2.28 for DWHP), and this was apparent even within the same category of occupation. For example, among nurses, the prevalence of DLBP ranged 4-fold: from 9.6% (95% CI 5.8%–14.8%) in Pakistan and 11.3% (95% CI 8.9%–14.1%) in Japan, to 37.7% (95% CI 31.3%–44.5%) in Costa Rica and 42.6% (95% CI 36.7%–48.6%) in Nicaragua. And for DWHP in office workers, rates varied more than 14-fold, ranging from 2.2% (95% CI 0.6%–5.6%) in Pakistan and 2.3% (95% CI 0.9%–4.6%) in Japan, to 31.3% (95% CI 25.9%–37.1%) in Brazil and 31.6% (95% CI 26.2%–37.3%) in Nicaragua. Overall, there was a strong correlation between the prevalence of pain at the 2 anatomical sites (Pearson correlation coefficient = 0.76), but with a tendency for DLBP to be relatively more common in nurses and DWHP in office workers.

Table 2 summarises the associations of disabling pain with personal risk factors. As might be expected from the differences in prevalence between nurses and office workers, DLBP was associated with occupational lifting, and DWHP with occupational use of a keyboard or other repetitive movements of the wrist/hand. DWHP was also more frequent among people who had heard of terms such as “RSI.” Otherwise, associations were similar for the 2 health outcomes, the highest PRRs being for report of 2 or more distressing somatic symptoms in the past week (2.10, 95% CI 1.88–2.33 for DLBP; and 2.24, 95% CI 1.99–2.52 for DWHP). In addition, both outcomes were more prevalent in women than men, at older ages, in current smokers, where work entailed time pressures, where there was a lack of support at work, when mental health was poor, and in people with adverse beliefs about the work-relatedness and prognosis of musculoskeletal pain.

**Fig. 2:**
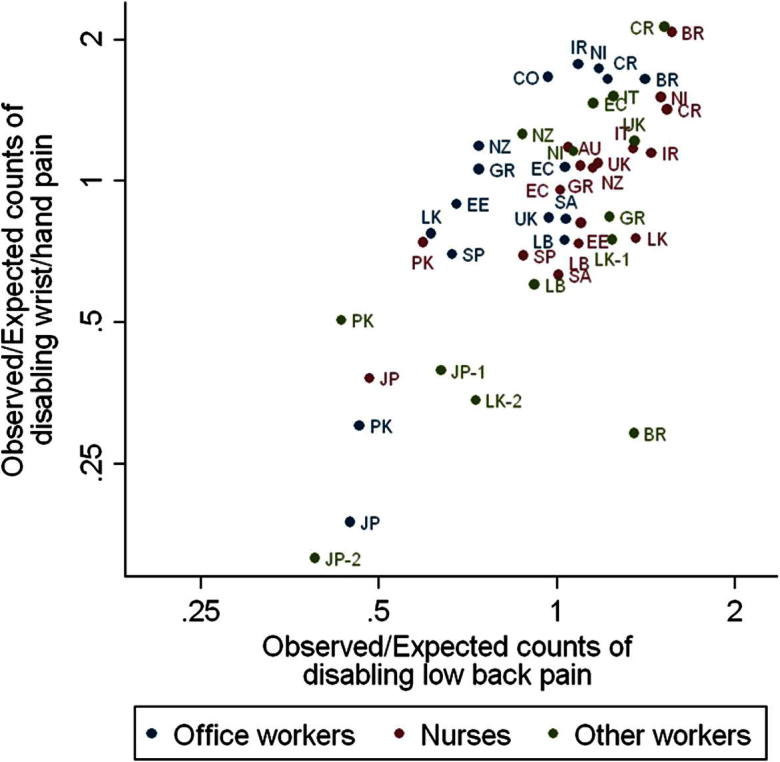
Ratios of observed counts of disabling low back and wrist/hand pain to those expected from the distribution of individual-level risk factors in each occupational group.

When account was taken of the personal risk factors listed in Table 2, the ratio of observed to expected cases still varied 4-fold among occupational groups for DLBP (geometric SD 1.45) and 14-fold for DWHP (geometric SD 1.82) (Fig. 2). Moreover, the correlation between rates of disabling pain at the 2 anatomical sites persisted (Pearson correlation coefficient = 0.72), as did the tendency for relatively more DLBP in nurses and relatively more DWHP in office workers.

Table 3 shows the associations of pain prevalence with group-level variables after account was taken of personal risk factors. In analyses that examined each group-level risk factor separately, the only variable significantly associated with DLBP was the group prevalence of knowing someone outside work with low back pain (PRR for 1 SD increase in prevalence 1.12, 95% CI 1.01–1.23). For DWHP, significant associations were observed with group prevalence of knowing someone outside work with arm pain (PRR for 1 SD increase in prevalence 1.40, 95% CI 1.25–1.58), group prevalence of adverse beliefs about the prognosis of arm pain (PRR for 1 SD increase in prevalence 1.23, 95% CI 1.10–1.38), and access to an occupational health service (PRR 1.47, 95% CI 1.05–2.05). When the associations with these 3 variables were mutually adjusted, risk estimates were somewhat reduced (PRRs 1.35, 1.10, and 1.33, respectively), but all remained statistically significant.

**Fig. 3:**
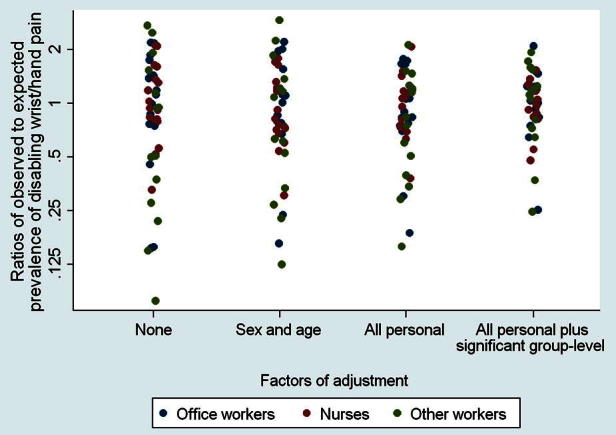
Ratios of observed to expected prevalence of disabling wrist/hand pain in occupational groups according to level of adjustment for risk factors.

After allowance for the significant group-level risk factors as well as personal risk factors, the variation in ratios of observed to expected cases of DWHP was further reduced, but remained 8-fold (geometric SD 1.57), as illustrated in Fig. 3. The occupational groups with the highest ratios were office workers in Colombia (2.1) and postal workers in the UK (1.9), and those with lowest ratios were sales workers in Japan (0.24) and office workers in Japan (0.25). Poisson regression with clustering by country as well as occupational group indicated that, of the residual variance attributable to occupational group and country, 92% was between-country.

To check for possible bias, we repeated analyses, including the method by which questionnaires were completed (interview, self-administered, or a combination) as an additional explanatory variable. Risk estimates were virtually unaltered. We also repeated analyses after exclusion of the 5 occupational groups in which the response rate was <50%. Again, results were essentially unchanged.

## 4 Discussion

Our study has demonstrated large variation among occupational groups internationally in the occurrence of disabling musculoskeletal illness. The variation applied even to groups carrying out similar occupational activities, and was only partially explained by the personal demographic, physical, and psychosocial risk factors examined. After allowance for personal risk factors, associations were found also with group awareness of others outside work with musculoskeletal pain, and for DWHP, with access to an occupational health service and adverse beliefs in the group about the prognosis of arm pain. However, even after these had been taken into account, there were still 8-fold differences in the prevalence of DWHP among occupational groups.

In comparisons of illness among countries, and particularly of subjective complaints, there is a possibility of bias because symptoms are understood differently in different languages and cultures. Thus, even with the care that we took in translation, the term “pain” may not have meant the same to all participants in our study. To reduce the potential for misinterpretation and bias, we focused on pain that made everyday activities difficult or impossible. Moreover, in some cases, large differences in prevalence were observed even among occupational groups from the same country who were questioned in the same language. For example, rates of DWHP in Brazilian office workers and nurses were some 15 times higher than those in other workers (sugar cane cutters) from Brazil. Thus, we do not think that the variation in reported pain prevalence can be explained simply by differences in the understanding of pain. The anatomical sites of interest were illustrated by diagrams, and are unlikely to have been misinterpreted systematically.

For practical reasons, it was necessary to administer the questionnaire by interview in some occupational groups (e.g., those with low literacy) and by self-completion in others (e.g., where they were geographically dispersed or their employers would not allow time for them to be interviewed). However, adjustment for the method by which questionnaires were answered did not materially alter our findings.

Another possible source of bias was the loss of subjects who declined to take part in the study. Although rates of participation were generally high, response rates in a few occupational groups were notably lower. However, exclusion of the 5 groups with response rates <50% did not significantly change the pattern of results.

Bias might also have arisen through differential healthy worker selection. If individuals with musculoskeletal disorders had been selected out of employment in some occupational groups because of their illness (or in some occupational groups were absent from work at the time when the study sample was recruited), spuriously low prevalence rates could have resulted. However, it seems highly unlikely that such selection would have occurred on a scale sufficient to explain the large differences in prevalence that were observed.

A further limitation was the relatively crude information that we obtained about occupational activities. Because of limited resources, we were unable to make detailed ergonomic assessments of working practices. If there were nondifferential errors in the ascertainment and characterisation of exposures, this would have tended to bias risk estimates towards the null, causing us to underestimate the contribution of physical activities to variations in prevalence among occupational groups. That said, observed differences in pain prevalence between nurses (who carried out more heavy lifting) and office workers (who carried out more work with computer keyboards) were much smaller than the differences among occupational groups carrying out similar activities in different countries (Fig. 1). Moreover, the variation in prevalence of DWHP that was unexplained after adjustment for measured risk factors did not appear to be related to occupational category. Thus, it seems unlikely that a more detailed and accurate assessment of individual activities would have accounted for substantially more of the variation among occupational groups.

Previous studies have also indicated major international variation in the prevalence of musculoskeletal pain [3,17]. However, findings cannot be compared directly with ours because they do not relate to specific occupational groups, the countries studied were largely different from those in our investigation, and the definition of pain outcomes differed from that which we used. Moreover, these studies did not explore risk factors that might explain the variation.

The associations that were found with personal risk factors, both physical and psychosocial, were much as would have been expected from previous research, including some analyses based on data from individual countries in the CUPID study [2,10,16,21]. Most notable was the elevated risk in people who tended to somatise, a finding that has been reported before, both for low back and wrist/hand pain, including in longitudinal studies in which somatising tendency predicted the future incidence and persistence of pain [12,19]. It would not be surprising if people who tended to worry about other common somatic symptoms were also more aware of, and more likely to report, musculoskeletal complaints. Associations were also observed with adverse beliefs about the work-relatedness and prognosis of musculoskeletal pain, and in the case of DWHP, with awareness of RSI or equivalent terms (Table 2). However, these were weaker. The patterns of association with psychosocial risk factors were similar for DLBP and DWHP, suggesting that similar psychological mechanisms contribute to both forms of illness.

A cross-sectional survey such as ours cannot establish the extent to which observed associations are causal. It may be, for example, that part of the association between disabling pain and low mood occurred because living with pain is depressing (although longitudinal studies indicate that if this does occur then it is not the full explanation [11]). Also, the presence of pain may make people more aware of, and more likely to report, occupational activities that are made difficult by the symptom. However, even with the assumption that all associations with personal risk factors were causal, those risk factors did not explain the major variation in pain prevalence among occupational groups. Furthermore, the persisting correlation between DWHP and DLBP after adjustment for personal risk factors (Fig. 2) suggests that whatever was responsible for the variation applied similarly to both health outcomes.

A particular strength of our study was its capacity to examine group-level, cultural, and socioeconomic influences on disabling pain while adjusting for personal risk factors at an individual level. However, with a total of only 47 occupational groups, we were concerned not to use too many degrees of freedom when analysing group-level risk factors. Thus, we first examined each group-level risk factor separately, and in our final model, retained only those that showed statistically significant associations when examined independently. As well as socioeconomic variables such as unemployment rate and social security support for the unemployed, we defined some group-level risk factors according to the prevalence of individual characteristics within each occupational group. In one case (knowing someone outside work with back or arm pain) there was a danger that the responses of individuals with pain might be biased by their illness (i.e., the occurrence of similar pain in other people would be brought to their attention because of their own symptoms). Thus, the group prevalence would be a more reliable measure. In other cases, we speculated that the group prevalence might reflect an environment that had an influence over and above that of the same characteristic in the individual.

The association of disabling pain with group awareness of people outside work with musculoskeletal symptoms may have occurred because nonoccupational risk factors increased the prevalence of such symptoms both in the occupational group and in the wider community from which it was drawn. Another possibility is that greater awareness of musculoskeletal pain in a community predisposed workers to develop symptoms through a nocebo effect, similar to that which has been proposed for chronic whiplash injury [15]. Against this, however, once account had been taken of the individual worker’s knowledge, DWHP was not related to group awareness of terms such as RSI.

The higher risk of DWHP where workers had access to an occupational health service may reflect a greater tendency to medicalise symptoms in these circumstances, especially if occupational health practitioners overstate the risks of musculoskeletal injury through work. However, it is also possible that in some cases occupational health services are engaged because of a high frequency of musculoskeletal disorders in a workforce.

Adjustment for significant group-level risk factors did reduce the differences in prevalence of DWHP among occupational groups, but fell a long way short of explaining the variation, which remained 8-fold. This indicates that there are other important determinants of common musculoskeletal complaints that were not adequately captured by the variables that we analysed.

Overall, our findings are consistent with the hypothesis that widespread awareness of musculoskeletal pain and adverse beliefs about it predispose to its occurrence in a workforce, but any such effect appears to be relatively small and did not account for major differences in prevalence that we observed. Nor could the variation be explained by well-established personal risk factors or by socioeconomic influences such as systems of compensation for work-related illness and injuries, and financial support for health-related incapacity for work.

## 5 Conflict of interest

The authors have no conflicts of interest to report.

## Figures and Tables

**Table 1:** Countries and occupational groups studied.

Country	Abbreviation	Occupational groups
Brazil	BR	Office workers, nurses, sugar cane cutters
Ecuador	EC	Office workers, nurse assistants, flower plantation workers
Colombia	CO	Office workers
Costa Rica	CR	Office workers, nurses, telephone call centre workers
Nicaragua	NI	Office workers, nurses, machine operators
UK	UK	Office workers, nurses, mail sorters
Spain	SP	Office workers, nurses
Italy	IT	Nurses, assembly line workers
Greece	GR	Office workers, nurses, postal clerks
Estonia	EE	Office workers, nurses
Lebanon	LB	Office workers, nurses, food production workers
Iran	IR	Office workers, nurses
Pakistan	PK	Office workers, nurses, mail sorters
Sri Lanka	LK	Office workers, nurses, mail sorters (other workers 1), sewing machinists (other workers 2)
Japan	JP	Office workers, nurses, transportation operatives (other workers 1), sales workers (other workers 2)
South Africa	SA	Office workers, nurses
Australia	AU	Nurses
New Zealand	NZ	Office workers, nurses, mail sorters

**Table 2:** Associations of disabling low back and wrist/hand pain with personal risk factors.

Risk factor		Disabling low back pain				Disabling wrist/hand pain		
	n	(%)	PRR^a^	(95% CI)	n	(%)	PRR^a^	(95% CI)
Sex								
Male	668	(15.4)	1		329	(7.6)	1	
Female	2071	(25.6)	1.31	(1.16–1.47)	1466	(18.2)	1.56	(1.37–1.78)

Age (years)								
20–29	507	(16.6)	1		341	(11.1)	1	
30–39	824	(20.7)	1.24	(1.13–1.37)	480	(12.1)	1.08	(0.93–1.26)
40–49	913	(26.4)	1.54	(1.38–1.72)	582	(16.9)	1.39	(1.15–1.67)
50–59	495	(25.5)	1.55	(1.39–1.72)	392	(20.2)	1.74	(1.36–2.22)

Smoking status								
Never smoked	1678	(21.4)	1		1164	(14.8)	1	
Ex-smoker	424	(23.8)	1.17	(1.07–1.28)	259	(14.5)	1.04	(0.94–1.16)
Current smoker	633	(23.0)	1.18	(1.06–1.31)	369	(13.4)	1.20	(1.06–1.35)
Missing	4	(10.3)	0.58	(0.28–1.22)	3	(7.7)	0.56	(0.23–1.35)

Activity in an average working day								
Lifting weights ⩾ 25 kg^b^	1117	(24.9)	1.16	(1.06–1.26)				
Use of keyboard or other repeated movements of wrist/hand for >4 h^c^					1559	(17.0)	1.63	(1.40–1.90)

Psychosocial aspects of work								
Work for >50 h per week	430	(16.1)	1.02	(0.94–1.11)	202	(7.6)	0.99	(0.85–1.15)
Time pressure at work	2218	(23.7)	1.20	(1.10–1.32)	1425	(15.3)	1.16	(1.04–1.29)
Incentives at work	785	(22.5)	1.03	(0.96–1.11)	500	(14.3)	0.96	(0.84–1.09)
Lack of support at work	821	(27.2)	1.13	(1.03–1.24)	570	(18.9)	1.12	(1.03–1.22)
Job dissatisfaction	598	(23.6)	1.07	(0.95–1.21)	362	(14.3)	1.11	(0.97–1.28)
Lack of job control	648	(24.3)	1.07	(0.98–1.16)	449	(16.8)	1.13	(1.03–1.25)
Job insecurity	940	(24.0)	1.12	(1.03–1.23)	541	(13.8)	0.95	(0.84–1.08)

Number of distressing somatic symptoms in past week								
0	1080	(14.6)	1		630	(8.5)	1	
1	661	(25.3)	1.47	(1.32–1.63)	446	(17.1)	1.53	(1.38–1.70)
2+	962	(42.1)	2.10	(1.88–2.33)	697	(30.5)	2.24	(1.99–2.52)
Missing	36	(28.3)	1.55	(1.09–2.20)	22	(17.3)	1.36	(0.92–2.02)

Mental health								
Good	797	(17.0)	1		563	(12.0)	1	
Intermediate	800	(21.3)	1.16	(1.08–1.26)	529	(14.1)	1.11	(0.97–1.27)
Poor	1128	(29.0)	1.42	(1.28–1.57)	696	(17.9)	1.27	(1.13–1.43)
Missing	14	(17.9)	0.87	(0.56–1.36)	7	(9.0)	0.74	(0.30–1.82)

Adverse beliefs about musculoskeletal pain^d^								
Work-relatedness	1174	(28.1)	1.26	(1.15–1.39)	738	(20.4)	1.37	(1.24–1.51)
Physical activity	518	(23.1)	1.01	(0.93–1.10)	178	(13.0)	0.83	(0.73–0.96)
Prognosis	510	(29.6)	1.27	(1.15–1.39)	271	(21.9)	1.16	(0.99–1.37)

Heard of “RSI” or equivalent^c^					1056	(15.5)	1.13	(1.03–1.24)

PRR, prevalence rate ratio; CI, confidence interval; RSI, repetitive strain injury.

a Mutually adjusted risk estimates derived from a single Poisson regression model for each outcome.

b Not included in model for disabling wrist/hand pain.

c Not included in model for disabling low back pain.

d About low back pain or arm pain according to the outcome.

**Table 3:** Associations of disabling low back and wrist/hand pain with group-level risk factors.

Risk factor	Number of occupational groups exposed	Level of exposure		Disabling low back pain		Disabling wrist/hand pain	
		Mean	SD	PRR^a^	(95% CI)	PRR^a^	(95% CI)
Group prevalence (%) of adverse beliefs about low back pain^b^							
Work-relatedness	47	0.32	0.20	0.98	(0.89–1.07)		
Physical activity	47	0.19	0.18	0.92	(0.84–1.01)		
Prognosis	47	0.12	0.08	1.04	(0.94–1.14)		

Group prevalence (%) of adverse beliefs about arm pain^b^							
Work-relatedness	47	0.30	0.18			1.06	(0.92–1.22)
Physical activity	47	0.12	0.12			0.89	(0.79–1.00)
Prognosis	47	0.10	0.07			1.23	(1.10–1.38)

Group prevalence (%) of knowing someone outside work with							
Low back pain^*b*^	47	0.59	0.14	1.12	(1.01–1.23)		
Arm pain^*b*^	47	0.41	0.12			1.40	(1.25–1.58)

Group prevalence (%) of having heard about “RSI” or equivalent^*b*^	47	0.52	0.25			1.05	(0.91–1.20)

Access to occupational health services (some or all workers)	38			1.32	(1.00–1.76)	1.47	(1.05–2.05)

Full sick pay in first 3 months absence	25			1.12	(0.92–1.35)	1.16	(0.87–1.55)

Financial support for ill-health retirement (sometimes or usually)	28			1.19	(0.94–1.51)	1.35	(0.94–1.94)

Social security for long-term unemployment	28			0.97	(0.78–1.19)	0.94	(0.69–1.27)

Compensation (any) for work-related musculoskeletal disorders of							
Back	38			1.20	(0.94–1.54)		
Arm	38					1.08	(0.79–1.48)

Unemployment rate ⩾ 10%	12			1.11	(0.91–1.34)	0.89	(0.66–1.20)

Payment for primary care (part or full)	19			1.01	(0.83–1.23)	1.13	(0.84–1.52)

PRR, prevalence rate ratio; CI, confidence interval; RSI, repetitive strain injury; SD, standard deviation.

a Each risk factor was examined independently in a separate Poisson regression model with adjustment for all of the risk factors in Table 2.

b Risk estimates are for an increase of one SD.
