# Supplementary material for: Disabling musculoskeletal pain in working populations: Is it the job, the person, or the culture?
Source: Pain. 2013 Jun;154(6):856–63. doi: 10.1016/j.pain.2013.02.008 (PMC3675684; doi:10.1016/j.pain.2013.02.008)
Supplement: Supplementary file 1 [file mmc1.doc]

# THE CUPID COLLABORATION

| **Name** | **Degrees** | **Affiliation** | **Email address** | **Contribution to study** |
| --- | --- | --- | --- | --- |
| **Brazil** |  |  |  |  |
| Vanda E Felli |  | School of Nursing, University of São Paulo, Brazil | [vandaeli@usp.br](mailto:vandaeli@usp.br) | Principal Investigator in Brazil and coordinator of data collection for nurses |
| Maria H Marziale | Prof | School of Nursing of Ribeirão Preto, University of São Paulo, Brazil | [marziale@usp.br](mailto:marziale@usp.br) | Coordinator of data collection in sugar cane cutters |
| Leila M Sarquis | Prof | Federal University of Paraná, Curitiba-PR, Brazil | [Leila.sarquis@ufpr.br](mailto:Leila.sarquis@ufpr.br) | Coordinator of data collection for office workers |
|  |  |  |  |  |
| **Ecuador** |  |  |  |  |
| Raúl Harari | MD PhD | Corporación para el Desarrollo de la Producción y el Medio Ambiente Laboral – IFA (Institute for the Development of Production and the Work Environment), Ecuador | [ifa@ifa.org.ec](mailto:ifa@ifa.org.ec) | Principal Investigator in Ecuador |
| Florencia Harari | MD | Corporación para el Desarrollo de la Producción y el Medio Ambiente Laboral – IFA (Institute for the Development of Production and the Work Environment), Ecuador | [ifa@ifa.org.ec](mailto:ifa@ifa.org.ec) | Coordinator of data collection for office workers |
| Rocío Freire MR | MSc | Corporación para el Desarrollo de la Producción y el Medio Ambiente Laboral – IFA (Institute for the Development of Production and the Work Environment), Ecuador | [ifa@ifa.org.ec](mailto:ifa@ifa.org.ec) | Coordinator of data collection for nurses |
| Natalia Harari Ms | Eng | Corporación para el Desarrollo de la Producción y el Medio Ambiente Laboral – IFA (Institute for the Development of Production and the Work Environment), Ecuador | [ifa@ifa.org.ec](mailto:ifa@ifa.org.ec) | Coordinator of data collection for flower plantation workers. |
|  |  |  |  |  |
| **Colombia** |  |  |  |  |
| Lope H Barrero Dr. | ScD, MSc, BE | School of Engineering, Pontificia Universidad Javeriana, Bogotá, Colombia | [lopehugo@javeriana.edu.co](mailto:lopehugo@javeriana.edu.co) | Principal Investigator in Colombia |
| Magda V Monroy Ms | BE | School of Engineering, Pontificia Universidad Javeriana, Bogotá, Colombia  Occupational Health Service, Parc de Salut MAR, Barcelona, Spain | [m.monroy@javeriana.edu.co](mailto:m.monroy@javeriana.edu.co) | Data collection |
| Leonardo A Quintana Dr. | PhD, MSc, BE | School of Engineering, Pontificia Universidad Javeriana, Bogotá, Colombia  Occupational Health Service, Parc de Salut MAR, Barcelona, Spain | [lquin@javeriana.edu.co](mailto:lquin@javeriana.edu.co) | Oversight of study design and conduct |
|  |  |  |  |  |
| **Costa Rica** |  |  |  |  |
| Marianela Rojas Ms | MSc | Institute for Studies on Toxic Substances (IRET), National University of Costa Rica | [mrojasg@una.ac.cr](mailto:mrojasg@una.ac.cr) | Coordinaotor of data collection and supervised data entry and checking |
| Patrica Monge dr | PhD | Institute for Studies on Toxic Substances (IRET), National University of Costa Rica | [pmonge@una.ac.cr](mailto:pmonge@una.ac.cr) | Data collection |
| Melania Chaverrri | Licentiate | Institute for Studies on Toxic Substances (IRET), National University of Costa Rica | [mchaverr@una.ac.cr](mailto:mchaverr@una.ac.cr) | Data collection |
| Freddy Brenes | MSc | Costa Rican Social Security System | [fbrenes@ccss.sa.cr](mailto:fbrenes@ccss.sa.cr) | Costa Rican Social SecuritySystem liaison and support of field work |
|  |  |  |  |  |
| **Nicaragua** |  |  |  |  |
| Aurora Aragón | PhD | Centre for Research in Health Work and Environment, Faculty of Medical Sciences, National Autonomous University of Nicaragua, León, Nicaragua | [aurora_aragon@yahoo.com](mailto:aurora_aragon@yahoo.com) | Coordination of data collection and supervided data entry and checking |
| Alberto Berríos | M.Sc | Centre for Research in Health Work and Environment, Faculty of Medical Sciences, National Autonomous University of Nicaragua, León, Nicaragua | [albertoinca@yahoo.es](mailto:albertoinca@yahoo.es) | Data dollection, data entry and checking |
|  |  |  |  |  |
| **USA** |  |  |  |  |
| Sarah A. Felknor | Dr PH | Center for Disease Control and Prevention/National Institute for Occupational Safety and Health, Atlanta, USA.  Southwest Center for Occupational and Environmental Health, The University of Texas Health Science Center at Houston School of Public Health, USA (now at CDC/NIOSH, Atlanta) | [sbf5@cdc.gov](mailto:sbf5@cdc.gov) | Joint Principal Investigator for Costa Rica and Nicaragua with input also to study design in Colombia |
| David Gimeno | PhD | Southwest Center for Occupational and Environmental Health, The University of Texas Health Science Center at Houston School of Public Health, USA | [David.Gimeno@uth.tmc.edu](mailto:David.Gimeno@uth.tmc.edu) | Joint Principal Investigator for Costa Rica and Nicaragua |
| Eduardo J. Salazar Vega | MPH, CPH | Southwest Center for Occupational and Environmental Health, The University of Texas Health Science Center at Houston School of Public Health, USA  School of Nursing, University of Sáo Paulo, Sáo Paulo, Brazil | [Eduardo.J.SalazarVega@uth.tmc.edu](mailto:Eduardo.J.SalazarVega@uth.tmc.edu) | Collation and checking of data from Costa Rica and Nicaragua |
|  |  |  |  |  |
| **UK** |  |  |  |  |
| David Coggon | FMedSci | MRC LIfecourse Epidemiology Unit, University of Southampton, UK | [dnc@mrc.soton.ac.uk](mailto:dnc@mrc.soton.ac.uk) | Principal Investigator for the CUPID study. Initiated and designed the study, oversaw statistical analysis, and wrote first draft of manuscript. |
| Georgia Ntani | MSc | MRC LIfecourse Epidemiology Unit, University of Southampton, UK | [gn@mrc.soton.ac.uk](mailto:gn@mrc.soton.ac.uk) | Carried out statistical analysis |
| Keith T Palmer | DM | MRC LIfecourse Epidemiology Unit, University of Southampton, UK | [ktp@mrc.soton.ac.uk](mailto:ktp@mrc.soton.ac.uk) | Contributed to design of international study |
| Ken Cox |  | MRC LIfecourse Epidemiology Unit, University of Southampton, UK | [kcc@mrc.soton.ac.uk](mailto:kcc@mrc.soton.ac.uk) | Collation and checking of international dataset |
| E Clare Harris | MSc | MRC LIfecourse Epidemiology Unit, University of Southampton, UK | [ech@mrc.soton.ac.uk](mailto:ech@mrc.soton.ac.uk) | Supervision of data collection in UK |
| Anna Cattrell |  | MRC Social, Genetic and Developmental Psychiatry Centre, Institute of Psychiatry, Kings College, London, UK | [anna.a.cattrell@kcl.ac.uk](mailto:anna.a.cattrell@kcl.ac.uk) | Data collection in UK |
|  |  |  |  |  |
| **Spain** |  |  |  |  |
| Sergio Vargas-Prada |  | Center for Research in Occupational Health (CiSAL), Pompeu Fabra University, Barcelona, Spain | [sergio.vargas@upf.edu](mailto:sergio.vargas@upf.edu) | Collection, preparation and checking of data in Spain |
| Consol Serra |  | 1. Center for Research in Occupational Health (CiSAL), Pompeu Fabra University, Barcelona, Spain. 2. CIBER of Epidemiology and Public Health (CIBERESP), Spain. 3. Occupational Health Department, Parc de Salut MAR, Barcelona, Spain. | [CSerraPujadas@parcdesalutmar.cat](mailto:CSerraPujadas@parcdesalutmar.cat) | Joint Principal Investigator in Spain |
| José Miguel Martínez |  | 1. Center for Research in Occupational Health (CiSAL), Pompeu Fabra University, Barcelona, Spain. 2. CIBER of Epidemiology and Public Health (CIBERESP), Spain. | [jmiguel.martinez@upf.edu](mailto:jmiguel.martinez@upf.edu) | Supervision of data collection and preparation in Spain |
| George Delclos | Dr. | 1. Center for Research in Occupational Health (CiSAL), Pompeu Fabra University, Barcelona, Spain. 2. CIBER of Epidemiology and Public Health (CIBERESP), Spain. 3. School of Public Health, University of Texas, Houston, USA | [George.Delclos@uth.tmc.edu](mailto:George.Delclos@uth.tmc.edu) | Supervision of data collection in Spain |
| Fernando G. Benavides |  | 1. Center for Research in Occupational Health (CiSAL), Pompeu Fabra University, Barcelona, Spain. 2. CIBER of Epidemiology and Public Health (CIBERESP), Spain. | [fernando.benavides@upf.edu](mailto:fernando.benavides@upf.edu) | Joint Principal Investigator in Spain |
|  |  |  |  |  |
| **Italy** |  |  |  |  |
| Matteo Bonzini Dr. | MD, PhD | Epidemiology and Preventive Medicine Research Center, University of Insubria, Varese, Italy | [matteo.bonzini@uninsubria.it](mailto:matteo.bonzini@uninsubria.it) | Principal Investigator for Italy |
| Michele Carugno  Dr. | MD | Department of Clinical Sciences and Community Health, Università degli Studi di Milano, Milan, Italy | [michele.carugno@gmail.com](mailto:michele.carugno@gmail.com) | Collection and checking of data in Italy |
| Marco M Ferrario | MD, PhD | Epidemiology and Preventive Medicine Research Center, University of Insubria, Varese, Italy | [marco.ferrario@uninsubria.it](mailto:marco.ferrario@uninsubria.it) | Contributed to design of data collection in Italy |
| Angela C Pesatori | MD, PhD | Department of Clinical Sciences and Community Health, Università degli Studi di Milano, and Fondazione Ca’ Granda Ospedale Maggiore Policlinico, Milan, Italy | [angela.pesatori@unimi.it](mailto:angela.pesatori@unimi.it) | Contributed to design of data collection in Italy |
|  |  |  |  |  |
| **Greece** |  |  |  |  |
| Eleni Solidaki | Dr. | Dept of Social Medicine, Medical School, University of Crete, Heraklion, Greece | [esolidak@edu.med.uoc.gr](mailto:esolidak@edu.med.uoc.gr) | Collection, preparation and checking of data in Greece |
| Leda Chatzi |  | Dept of Social Medicine, Medical School, University of Crete, Heraklion, Greece | [lchatzi@med.uoc.gr](mailto:lchatzi@med.uoc.gr) |  |
| Panos Bitsios |  | Dept of Psychiatry, Medical School, University of Crete, Heraklion, Greece | [pbitsios@med.uoc.gr](mailto:pbitsios@med.uoc.gr) |  |
| Manolis Kogevinas | Prof. | 1. Centre for Research in Environmental Epidemiology (CREAL), Barcelona, Spain 2. IMIM (Hospital del Mar Research Institute), Barcelona, Spain 3. CIBER Epidemiologia y Salud Pública (CIBERESP), Barcelona, Spain | [kogevinas@creal.cat](mailto:kogevinas@creal.cat) | Principal Investigator for data collection in Greece |
|  |  |  |  |  |
| **Estonia** |  |  |  |  |
| Eda Merisalu Dr. |  | University of Tartu, Estonia | [eda.merisalu@ut.ee](mailto:eda.merisalu@ut.ee) | Principal Investigator in Estonia. |
| Kristel Oha Ms |  | North Estonia Medical Centre, Tallinn, Estonia | [kristel.oha@ut.ee](mailto:kristel.oha@ut.ee) | Carried out data collection in Estonia |
| Tiina Freimann Ms | MSc | Department of Public Health, University of Tartu; Tartu University Hospital | [tiina.feimann@kliinikum.ee](mailto:tiina.feimann@kliinikum.ee) | Data preparation in Estonia |
| Tuuli Sirk Ms | MSc | Põlva Hospital | [tuuli.sirk@gmail.com](mailto:tuuli.sirk@gmail.com) | Data collection and preparation in Estonia |
|  |  |  |  |  |
| **Lebanon** |  |  |  |  |
| Rima R. Habib | Ph.D | Department of Environmental Health,  American University of Beirut, Lebanon | [Rima.habib@aub.edu.lb](mailto:Rima.habib@aub.edu.lb) | Principal Investigator in Lebanon |
|  |  |  |  |  |
| **Iran** |  |  |  |  |
| Farideh Sadeghian  Ms | MSC | Shahroud University of Medical Sciences, Shahroud, Iran | [farsadeghian@googlemail.com](mailto:farsadeghian@googlemail.com) | Principal Investigator in Iran |
| Ali Sadeghian | MD | Klinikum Leverkusen, Leverkusen ,Germany | [alisadeghianmd@Yahoo.com](mailto:alisadeghianmd@Yahoo.com) | Oversight of data collection |
|  |  |  |  |  |
| **Pakistan** |  |  |  |  |
| M Masood Kadir Dr. | MBBS, MPH, MOHS, FCPS | Department of Community Health Sciences, Aga Khan University, Karachi, Pakistan | [masood.kadir@aku.edu](mailto:masood.kadir@aku.edu) | Principal Investigator in Pakistan |
|  |  |  |  |  |
| **Sri Lanka** |  |  |  |  |
| Sudath SP Warnakulasuriya | MScN | Department of Medical Education and Health Sciences, Faculty of Medical Sciences, University of Sri Jayewardenepura, Sri Lanka | [ashara96@yahoo.com](mailto:ashara96@yahoo.com) | Collection, preparation and checking of data in Sri Lanka |
| Roshini J Peiris-John | PhD | 1. Department of Physiology, Faculty of Medical Sciences, University of Sri Jayewardenepura, Sri Lanka 2. Section of Epidemiology and Biostatistics, School of Population Health, Faculty of Medical and Health Sciences, University of Auckland, New Zealand | [roshipj@hotmail.com](mailto:roshipj@hotmail.com) | Supervision of data collection in Sri Lanka |
| Nalini Sathiakumar | DrPH | Department of Epidemiology, School of Public Health, University of Alabama at Birmingham, USA | [NSathiakumar@ms.soph.uab.edu](mailto:NSathiakumar@ms.soph.uab.edu) | Supervision of data collection in Sri Lanka |
| A Rajitha Wickremasinghe | PhD | Faculty of Medicine, University of Kelaniya, Sri Lanka | [arwicks@sltnet.lk](mailto:arwicks@sltnet.lk) | Principal Investigator for Sri Lanka |
|  |  |  |  |  |
| **Japan** |  |  |  |  |
| Ko Matsudaira  Dr |  | Clinical Research Centre for Occupational Musculoskeletal Disorders, Kanto Rosai Hospital, Kawasaki, Japan | [kohart801@gmail.com](mailto:kohart801@gmail.com) | Principal Investigator for Japan |
| Noriko Yoshimura  Prof |  | Department of Joint Disease Research, University of Tokyo, Tokyo, Japan | [yoshimuran-ort@h.u-tokyo.ac.jp](mailto:yoshimuran-ort@h.u-tokyo.ac.jp) | Contributed to design of data collection in Japan, including translation of questionnaire |
|  |  |  |  |  |
| **South Africa** |  |  |  |  |
| Busisiwe Nyantumbu Ms | MSc (Med) | 1. National Institute for Occupational Health, National Health Laboratory Service, South Africa 2. University of Witwatersrand, South Africa | [busisiwe.nyantumbu@nioh.nhls.ac.za](mailto:busisiwe.nyantumbu@nioh.nhls.ac.za) | Collection, preparation and checking of data in South Africa |
| Danuta Kielkowski | PhD | School of Public Health, Witwatersrand, South Africa | [Danuta.kielkowski@nioh.nhls.ac.za](mailto:Danuta.kielkowski@nioh.nhls.ac.za) | Principal Investigator for South Africa |
|  |  |  |  |  |
| **Australia** |  |  |  |  |
| Malcolm R Sim  Prof | BMedSc, MBBS MSc GDipOccHyg  PhD FAFOEM FAFPHM FFOM | Department of Epidemiology and Preventive Medicine, School of Public Health and Preventive Medicine, Monash University, The Alfred Centre, Melbourne, Victoria, Australia | [malcolm.sim@monash.edu](mailto:malcolm.sim@monash.edu) | Principal investigator for Australia |
| Helen L Kelsall | MBBS MPH MHlthSc PhD FAFPHM | Department of Epidemiology and Preventive Medicine, School of Public Health and Preventive Medicine, Monash University, The Alfred Centre, Melbourne, Victoria, Australia | [helen.kelsall@monash.edu](mailto:helen.kelsall@monash.edu) | Coordinated collection, preparation and checking of data in Australia |
| Victor CW Hoe | MBBS MPH MPH (OH) MEng (SHE) | 1. Department of Epidemiology and Preventive Medicine, School of Public Health and Preventive Medicine, Monash University, The Alfred Centre, Melbourne, Victoria, Australia 2. Centre for Occupational and Environmental Health, Department of Social and Preventive Medicine, Faculty of Medicine, University of Malaya, 50603 Kuala Lumpur, Malaysia | [drvictorhoe@gmail.com](mailto:drvictorhoe@gmail.com) | Contributed to collection, preparation and checking of data in Australia |
| Donna M Urquhart | BPhysio (Hons), PhD | Department of Epidemiology and Preventive Medicine, School of Public Health and Preventive Medicine, Monash University, The Alfred Centre, Melbourne, Victoria, Australia | [donna.urquhart@monash.edu](mailto:donna.urquhart@monash.edu) | Contributed to questionnaire development and data collection in Australia |
|  |  |  |  |  |
| **New Zealand** |  |  |  |  |
| Helen Harcombe |  | Department of Preventive and Social Medicine, University of Otago, New Zealand | [helen.harcombe@otago.ac.nz](mailto:helen.harcombe@otago.ac.nz) | Contributed to data collection, critical revision of the manuscript for important intellectual content and obtaining funding in New Zealand. |
| Sarah Derrett |  | Injury prevention Research Unit, Department of Preventive and Social Medicine, University of Otago, New Zealand | [sarah.derrett@ipru.otago.ac.nz](mailto:sarah.derrett@ipru.otago.ac.nz) | Contributed to collection. Analysis and interpretation of data, critical revision of the manuscript for important intellectual content and obtaining funding in New Zealand. |
| David McBride |  | Department of Preventive and Social Medicine, University of Otago, New Zealand | [david.mcbride@otago.ac.nz](mailto:david.mcbride@otago.ac.nz) | Contributed to critical revision of the manuscript for important intellectual content and obtaining funding in New Zealand. |
| Peter Herbison |  | Department of Preventive and Social Medicine, University of Otago, New Zealand | [peter.herbison@otago.ac.nz](mailto:peter.herbison@otago.ac.nz) | Contributed to analysis and interpretation of data, critical revision of the manuscript for important intellectual content, statistical analysis and supervision in New Zealand |
| Andrew Gray |  | Department of Preventive and Social Medicine, University of Otago, New Zealand | [andrew.gray@otago.ac.nz](mailto:andrew.gray@otago.ac.nz) | Contributed to acquisition of data, critical revision of the manuscript for important intellectual content and supervision in New Zealand. |

In addition, all authors contributed to the revision and finalisation of the draft manuscript.
